# The Transcription Factor NFAT5 Is Required for Cyclin Expression and Cell Cycle Progression in Cells Exposed to Hypertonic Stress

**DOI:** 10.1371/journal.pone.0005245

**Published:** 2009-04-21

**Authors:** Katherine Drews-Elger, M. Carmen Ortells, Anjana Rao, Cristina López-Rodriguez, Jose Aramburu

**Affiliations:** 1 Departament de Ciències Experimentals i de la Salut, Universitat Pompeu Fabra, and Barcelona Biomedical Research Park (PRBB), Barcelona, Spain; 2 Immune Disease Institute and Department of Pathology, Harvard Medical School, Boston, Massachusetts, United States of America; New York University School of Medicine, United States of America

## Abstract

**Background:**

Hypertonicity can perturb cellular functions, induce DNA damage-like responses and inhibit proliferation. The transcription factor NFAT5 induces osmoprotective gene products that allow cells to adapt to sustained hypertonic conditions. Although it is known that NFAT5-deficient lymphocytes and renal medullary cells have reduced proliferative capacity and viability under hypertonic stress, less is understood about the contribution of this factor to DNA damage responses and cell cycle regulation.

**Methodology/Principal Findings:**

We have generated conditional knockout mice to obtain NFAT5^−/−^ T lymphocytes, which we used as a model of proliferating cells to study NFAT5-dependent responses. We show that hypertonicity triggered an early, NFAT5-independent, genotoxic stress-like response with induction of p53, p21 and GADD45, downregulation of cyclins, and cell cycle arrest. This was followed by an NFAT5-dependent adaptive phase in wild-type cells, which induced an osmoprotective gene expression program, downregulated stress markers, resumed cyclin expression and proliferation, and displayed enhanced NFAT5 transcriptional activity in S and G2/M. In contrast, NFAT5^−/−^ cells failed to induce osmoprotective genes and exhibited poorer viability. Although surviving NFAT5^−/−^ cells downregulated genotoxic stress markers, they underwent cell cycle arrest in G1/S and G2/M, which was associated with reduced expression of cyclins E1, A2 and B1. We also show that pathologic hypertonicity levels, as occurring in plasma of patients and animal models of osmoregulatory disorders, inhibited the induction of cyclins and aurora B kinase in response to T cell receptor stimulation in fresh NFAT5^−/−^ lymphocytes.

**Conclusions/Significance:**

We conclude that NFAT5 facilitates cell proliferation under hypertonic conditions by inducing an osmoadaptive response that enables cells to express fundamental regulators needed for cell cycle progression.

## Introduction

Hypertonic stress occurs in cells exposed to elevated extracellular tonicity, which causes the uptake of Na^+^ and other ions as a rapid response mechanism to maintain cell volume. Since the resulting increase in intracellular ionic strength is harmful for the function of cellular components, cells synthesize chaperones, such as Hsp70, as well as enzymes and transporters whose collective function is to increase the intracellular concentration of compatible organic osmolytes to normalize the ionic strength of the intracellular fluid [1], [2]. Hypertonicity can be harmful for cells, as it can induce double-strand DNA breaks, interfere with DNA repair, exacerbate the effect of genotoxic agents such as UV and ionizing radiation, inhibit proliferation and induce apoptosis (reviewed in [2]). In this regard, studies in immortalized renal medullary cells and other cell lines have shown that hypertonic stress can activate proteins involved in DNA damage sensing and checkpoint activation, such as Nbs1 [3], ATM, p53, Chk2 [3], [4], and GADD45 [5].

The transcription factor NFAT5/TonEBP is a major activator of the expression of osmoprotective gene products in mammalian cells. This protein belongs to the Rel family, which also comprises NF-κB and the calcineurin-dependent NFATc proteins [6]–[8]. NFAT5 is activated by hypertonic stress, by mechanisms involving DNA-damage responsive and stress-activated kinases such as ATM [9], DNA-PKc [10] and p38 [11], [12]. NFAT5-regulated osmoprotective gene products include, among others, the chaperones Hsp70 [13] and Hspa4l/Osp94 [14], and enzymes and transporters that increase the intracellular concentration of compatible osmolytes, such as aldose reductase (AR), the Na^+^/Cl^−^-coupled betaine/γ-aminobutyric acid transporter (BGT1), the Na^+^/myo-inositol cotransporter (SMIT) [6], the Na^+^ and Cl^−^-dependent taurine transporter (TauT) [15], the UT-A urea transporter [16], [17], and the sodium-dependent neutral aminoacid transporter (ATA2, SNAT2) [18].

It has been found that a wide variety of mammalian cell types have the ability to respond and adapt to hypertonic conditions. Overall, NFAT5 has been shown to be functional in response to hypertonicity in cell types as diverse as renal medullary cells [19]–[21], embryonic fibroblasts [20], neurons [22], lymphocytes [18], [23], [24], macrophages [25], myoblasts [26] and cardiomyocytes [27]. The importance of NFAT5 for the adaptive response of primary cells to osmotic stress has been addressed in mouse models of NFAT5 deficiency. Our previous work showed that NFAT5-null mice suffered severe atrophy and cellular loss in the renal medulla due to deficient expression of the osmoprotective gene products aldose reductase, BGT1 and SMIT [20]. Another study showed that overexpression of a dominant negative NFAT5 transgene in kidney collecting tubules impaired the expression of UT-A and aquaporin 2 and also caused atrophy of the renal medulla [19]. The same group later reported that expression of this dominant negative in non-proliferating, terminally differentiated eye lens fiber cells caused apoptosis and accumulation of the DNA damage markers p53 and phospho-Chk2 [28]. Besides these models, the Ho laboratory showed that impairment of NFAT5 function in T cells caused a decrease in their viability and proliferative capacity under hypertonic stress [18], [24].

The study of the role of NFAT5 in specific types of primary cells has been hindered by the severe phenotype of NFAT5-deficient mice, since only a small proportion survive after birth, and those that do manifest pronounced renal atrophy and growth defects [20]. To circumvent these problems, we generated NFAT5^Flox/Flox^ mice which could be used to inactivate NFAT5 in a tissue-specific manner. We have crossed the NFAT5^Flox/Flox^ mice to CD4-Cre animals to obtain mice with a selective deletion of NFAT5 in mature T cells, and analyzed the role of this factor in the hypertonic stress response and cell cycle regulation in T lymphocytes. We have used lymphocytes as a model of non-transformed, proliferating cells since previous work by us and others has shown that they regulate NFAT5 comparably to other cell types [18], [23]–[25]. Our results show that hypertonicity elicited an early, NFAT5-independent, genotoxic stress response and cell cycle arrest in proliferating lymphocytes, which was followed by an NFAT5-dependent phase in which cells induced osmoprotective gene products, downregulated genotoxic stress markers and reactivated the cell cycle. Lack of NFAT5 did not substantially affect the DNA damage-like response, but impaired the expression of osmoprotective genes and caused cell cycle arrest associated with defective expression of G1, S and G2 cyclins and aurora B kinase.

## Results

### 1) Defective induction of osmoprotective genes and cell cycle arrest in NFAT5^−/−^ T lymphocytes exposed to hypertonicity

We were interested in analyzing how the deficiency of NFAT5 in proliferating cells affected hypertonicity-regulated processes such as the induction of genotoxic stress-like responses and the cell cycle. Since NFAT5 deficiency impairs the survival and proliferation of T cells [18], [24], and lymphocytes regulate NFAT5 comparably to other cell types [23], [25], we studied lymphocytes as a model of non-transformed proliferating cells. To overcome the severe viability problems encountered with NFAT5-null mice [20], [24], we generated conditional NFAT5-knockout mice which lacked NFAT5 only in mature T cells (see Methods and Supporting information Figs. S1 and S2 for a detailed description of these mice). Mice lacking NFAT5 specifically in T cells had no apparent defects compared to wild-type animals in terms of viability and development and, in contrast to previous models of NFAT5 deficiency [18], [24], had normal numbers and proportions of T cells *in vivo* (Fig. S2).

**Figure 1:**
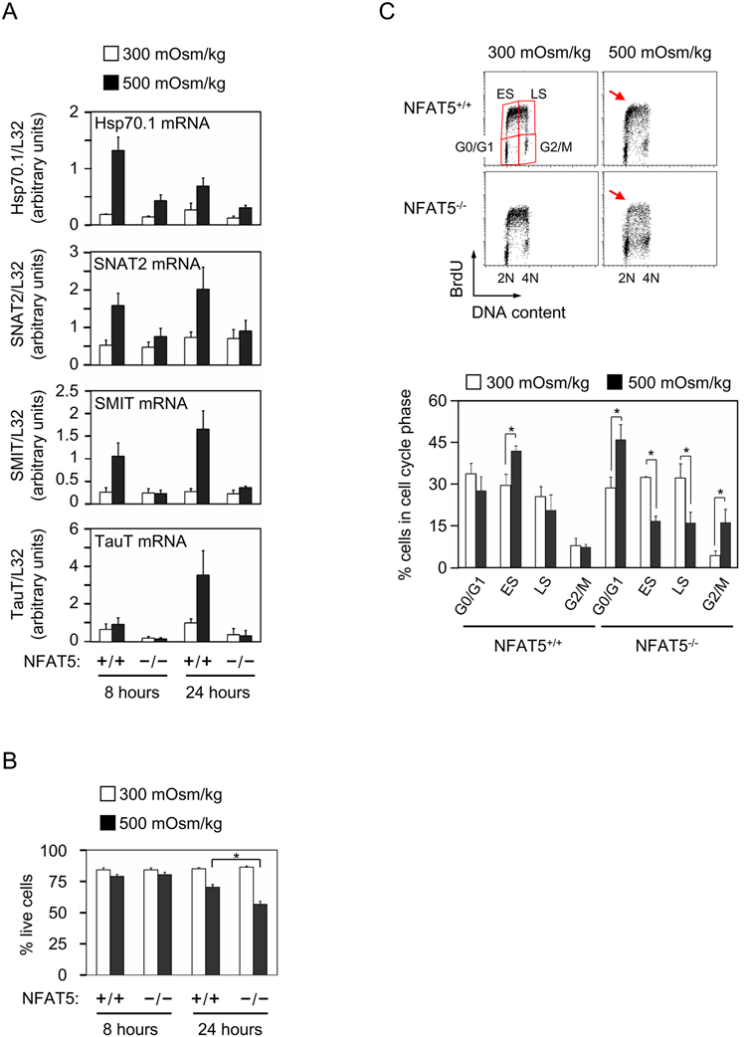
Induction of osmoprotective gene products, viability and cell cycle in proliferating NFAT5^−/−^ lymphocytes under hypertonic stress. A) RNA was isolated from NFAT5^+/+^ and NFAT5^−/−^ proliferating T cells that were either maintained in isotonic conditions (300 mOsm/kg) or switched to hypertonic medium (500 mOsm/kg) for 8 and 24 hours. Relative mRNA abundance for each gene was determined by RT-qPCR and values were normalized to their respective L32 mRNA levels (bars are mean±SEM of four independent experiments). B) Percentage of viable cells in cultures of NFAT5^+/+^ and NFAT5^−/−^ proliferating T cells after 8 or 24 hours in isotonic (300 mOsm/kg) or hypertonic conditions (500 mOsm/kg). Bars represent the mean±SEM of five independent experiments, (* = p<0.05). C) Proliferating lymphocytes growing in isotonic medium or switched to hypertonic medium during 24 hours were pulsed with BrdU during the last 30 minutes of culture, then fixed and analyzed by flow cytometry. The upper panel depicts one representative experiment showing BrdU incorporation plotted against DNA content, and the lower panel represents the results (mean±SEM) from three independent experiments (* = p<0.05).

We analyzed the induction of osmoprotective gene products in response to hypertonicity in proliferating T cells obtained by culturing splenocytes with the mitogen concanavalin A plus IL-2 during 72 hours. Cells were then cultured during 8 or 24 additional hours in either isotonic medium (300 mOsm/kg), or subjected to hypertonic conditions (500 mOsm/kg). Hypertonicity induced, in an NFAT5-dependent manner, the expression of Hsp70.1, the Na^+^/myo-inositol cotransporter (SMIT), the sodium-dependent neutral aminoacid transporter 2 (SNAT2), and the Na^+^ and Cl^−^-dependent taurine transporter (TauT) (Fig. 1A). Of these, Hsp70.1 was induced the earliest and was downregulated by 24 hours, while SMIT and SNAT2 showed sustained expression at 8 and 24 hours, and TauT was expressed at later times (24 hours). These results showed that NFAT5 activated an osmoprotective program in T cells that included gene products known to be induced by hypertonicity in other cells, indicating that different cell types utilize similar NFAT5-regulated mechanisms to respond to osmotic stress.

In parallel experiments, we observed that NFAT5^−/−^ T cells exposed to hypertonicity during 24 hours exhibited poorer viability (56%) than wild-type cells (70%) (Fig. 1B), and those surviving displayed cell cycle defects, with cultures having a lower proportion of cells in S, and a greater accumulation in G1 and G2/M (Fig. 1C). The decrease in BrdU uptake in NFAT5^−/−^ cells indicated defective DNA replication, which was in agreement with results by Go et al. showing reduced 3H-Thy incorporation in NFAT5-deficient lymphocytes under osmotic stress [24]. We observed that cell cycle defects in NFAT5^−/−^ T cells were more evident at later times (24 hours) than in the first 8 hours, in which both wild-type and NFAT5^−/−^ T cells showed a comparably mild viability loss and a similarly pronounced cell cycle arrest with enhanced accumulation in S and G2/M relative to G0/G1 (Fig. S3). Both wild-type and NFAT5^−/−^ cells had the same viability and cell cycle profile in isotonic conditions (Fig. 1B, 1C and S3), indicating that lack of NFAT5 did not affect the expansion and proliferative capacity of T cells in the absence of hypertonic stress. Altogether, these results indicated that hypertonicity triggered a rapid cell cycle arrest in both wild-type and NFAT5^−/−^ T cells, but while the former induced osmoprotective gene products and resumed proliferation with only a moderate viability loss, NFAT5^−/−^ cells had poorer viability, and those surviving exhibited cell cycle defects. At this point, we explored two potential scenarios to address the causes underlying the cell cycle arrest of NFAT5^−/−^ cells: one, we asked whether these cells displayed enhanced markers of genotoxic stress; and two, we analyzed how hypertonicity affected the expression of cell cycle regulators in wild-type and NFAT5^−/−^ lymphocytes.

### 2) Induction of p53 and markers of genotoxic stress in hypertonicity-treated NFAT5^−/−^ cells

**Figure 2:**
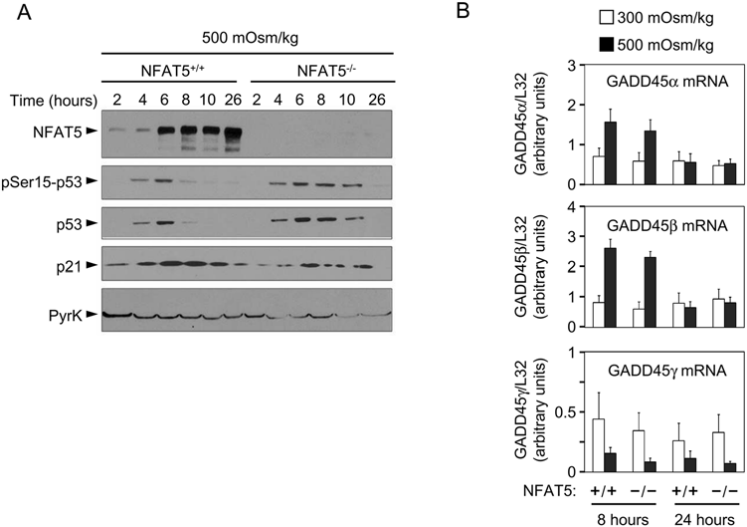
Induction of p53, p21 and GADD45 in proliferating NFAT5^−/−^ lymphocytes exposed to hypertonic stress. A) Western blot shows the time course of p53-Ser15 phosphorylation, accumulation of total p53 and induction of p21 in NFAT5^+/+^ and NFAT5^−/−^ cells in response to hypertonicity. The experiment shown is representative of three independently performed (see Fig. S4). B) RNA was isolated from NFAT5^+/+^ and NFAT5^−/−^ proliferating T cells that were either maintained in isotonic conditions (300 mOsm/kg) or switched to hypertonic medium (500 mOsm/kg) for 8 and 24 hours. Relative mRNA abundance for each GADD45 isoform was determined by RT-qPCR and values were normalized to their respective L32 mRNA levels (bars are mean±SEM of four independent experiments).

We analyzed the induction of stress markers associated with DNA damage responses. As shown in Fig. 2A, both NFAT5^+/+^ and NFAT5^−/−^ cells displayed a comparable activation of p53 early after exposure to hypertonicity (4–6 hours). This response was downregulated in wild-type cells by 8 hours, but persisted in NFAT5^−/−^ cells up to at least 10 hours, although eventually it subsided by 24 hours, and only a small proportion (∼7%) of live NFAT5^−/−^ cells had phospho-Ser15-p53 after 24 hours, as detected by intracellular staining (Fig. S4A). Consistent with the activation of p53, p21 was rapidly upregulated in response to hypertonic stress in both wild-type and NFAT5^−/−^ cells (Fig. 2A and S4B). Of note, although induction of p53 preceded the increase in NFAT5 expression, this was p53-independent, whereas induction of p21 required p53 (Fig. S4C). Despite the more prolonged induction of p53 in NFAT5^−/−^ lymphocytes, these cells did not accumulate greater amounts of the p53 targets p21 and GADD45α and β (Fig. 2 and Fig. S4). GADD45γ was comparably downregulated by osmotic stress in both cell types (Fig. 2B). These results showed that induction of at least some p53-dependent cell cycle repressors in response to hypertonicity was very similar in wild-type and NFAT5-deficient cells, suggesting that the persistent cell cycle arrest observed in the latter might not depend on p53.

**Figure 3:**
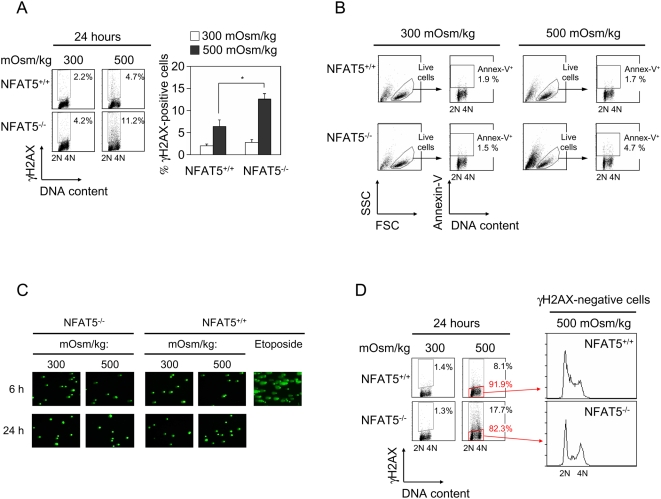
Analysis of DNA damage markers in proliferating NFAT5^−/−^ lymphocytes exposed to hypertonic stress. A) Dot plots representing γH2AX (H2AX phosphorylated in Ser 139) and DNA content in live NFAT5^+/+^ and NFAT5^−/−^ lymphocytes after 24 hours in isotonic or hypertonic conditions. Bars on the right represent the percentage of γH2AX^+^ cells after 24 hours in isotonic or hypertonic medium (values are the mean±SEM of seven independent experiments; * = p<0.05). B) T cells grown in isotonic or hypertonic conditions during 24 hours were stained with the DNA dye Hoechst 33342 and annexin-V-Fluos, and analyzed by flow cytometry. The experiment shown is representative of three independently performed. C) Single-cell alkaline gel electrophoresis assay (comet assay) done on the population of live cells isolated from cultures of NFAT5^−/−^ and wild-type cells after 6 or 24 hours in isotonic or hypertonic conditions. Etoposide-treated wild-type cells are included as a positive control. The experiment shown is representative of three independently performed. D) Cell cycle distribution of viable, γH2AX-negative cells after 24 hours in hypertonic medium (representative of seven independent experiments).

We next assessed whether NFAT5^−/−^ cells had a greater extent of DNA damage than wild-type cells under hypertonic conditions. Detection of γH2AX, a sensitive marker of double strand DNA breaks, showed only a moderately higher proportion of γH2AX^+^ cells in NFAT5^−/−^ cultures (11.6%) than in wild-type ones (5.2%) after 24 hours of stress (Fig. 3A). Also, a small fraction (<5%) of NFAT5^−/−^ cells were undergoing apoptosis, as shown by annexin-V staining (Fig. 3B). These results indicated that the population of NFAT5^−/−^ cells gated as alive contained a very low percentage of cells exhibiting markers of either enhanced genotoxic stress or apoptosis. Consistent with this result, analysis of DNA breaks by alkaline comet assay did not reveal a greater extent of DNA damage in live NFAT5^−/−^ cells than in wild-type ones (Fig. 3C). In addition, NFAT5-deficient lymphocytes that did not have phosphorylated H2AX still exhibited features of cell cycle arrest (fewer cells in S phase and accumulation in G2/M) (Fig. 3D). Altogether, these results suggested that the cell cycle arrest of NFAT5^−/−^ cells exposed to hypertonicity was not associated with a generalized genotoxic stress response.

### 3) Defective expression of cyclins in hypertonicity-treated NFAT5^−/−^ cells

**Figure 4:**
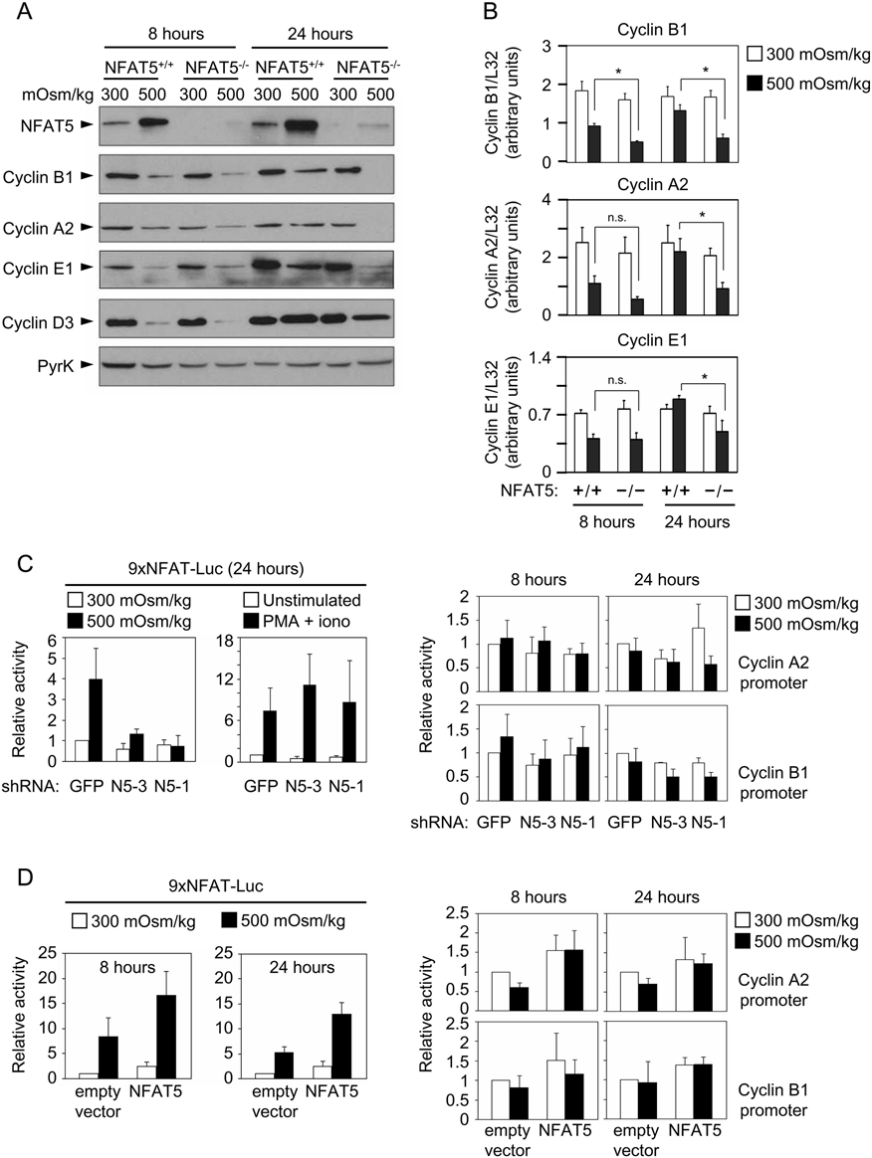
Expression of cyclins in proliferating NFAT5^−/−^ T cells upon exposure to hypertonic conditions. A) Expression of cyclins D3, E1, A2 and B1 was analyzed by Western blot in lysates of proliferating NFAT5^+/+^ and NFAT5^−/−^ T cells after 8 and 24 hours of hypertonicity treatment. Pyruvate kinase (PyrK) is shown as protein loading control. The result is representative of at least four independent experiments (see Fig. S5). B) mRNA abundance of cyclins was analyzed by RT-qPCR in proliferating NFAT5^+/+^ and NFAT5^−/−^ T cells subjected to hypertonicity for 8 and 24 hours. Values were normalized to L32 mRNA levels in each respective sample (bars are the mean±SEM of four independent experiments; * = p<0.05; n.s. = not statistically significant). C) Effect of inhibiting NFAT5 on the activity of cyclin A2 and cyclin B1 promoters. Jurkat T cells cotransfected with the indicated shRNA vectors, reporter constructs and pTK-Renilla were placed in fresh medium 36 hours after transfection, and either left untreated, subjected to hypertonicity, or stimulated with PMA plus ionomycin (PMA+iono) as indicated. The left panel (9×NFAT-Luc) shows the effect of the respective shRNAs on the NFAT5-dependent activation of the control reporter 9×NFAT-Luc by hypertonicity or its NFAT5-independent activation by PMA plus ionomycin. The right panel shows the effect of shRNAs on the activity of cyclin A2 and B1 promoter constructs after 8 and 24 hours in isotonic or hypertonic conditions. Luciferase activity was normalized to that of the pTK-Renilla reporter. Graphs show the mean±SD of four independent experiments. D) Jurkat cells cotransfected with reporter constructs and either empty vector (CMV-HA) or an NFAT5-expressing vector were left untreated or subjected to hypertonicity during 8 or 24 hours. Luciferase activity was normalized to that of the pTK-Renilla reporter. Graphs show the mean±SD of three independent experiments.

We analyzed the expression of several cyclins relevant for cell cycle progression. A short exposure to hypertonic stress (8 hours) had similar effects on the expression of different cyclins in wild-type and NFAT5^−/−^ lymphocytes: cyclin D3 was downregulated in both cell types in six out of six independent experiments, cyclin E1 in two out of five, and cyclin B1 in three out of seven independent cultures tested for each wild-type and NFAT5^−/−^ T cells, whereas cyclin A2 was not affected in the majority of experiments (Fig. 4A and S5). However, after 24 hours in hypertonic medium, wild-type and NFAT5^−/−^ cells displayed distinct differences. Whereas wild-type lymphocytes maintained the expression of cyclins E1, A2 and B1, NFAT5^−/−^ cells had substantially reduced levels of these cyclins (Fig. 4A and Fig. S5), though they were able to express cyclin D3. We analyzed whether downregulation of cyclins was associated with a decrease in their mRNA levels (Fig. 4B). The amount of mRNA for cyclins A2 and B1 was substantially decreased (by 60–70%) in both wild-type and NFAT5^−/−^ lymphocytes after 8 hours of hypertonicity treatment. By 24 hours, wild-type lymphocytes had recovered similar cyclin mRNA levels as those of cells grown in isotonic medium, whereas NFAT5^−/−^ cells did not. With regard to cyclin E1, its protein levels were much more reduced than its mRNA abundance in NFAT5^−/−^ cells, suggesting that its downregulation might involve defective protein synthesis and/or enhanced degradation. We next analyzed whether the lack of NFAT5 affected the activity of cyclin A2 and B1 promoters. Jurkat T cells were transfected with NFAT5-specific shRNAs and reporter constructs driven by cyclin promoters, and then cultured in isotonic or hypertonic conditions during 8 or 24 hours. Since the activity of at least the cyclin A2 promoter is regulated by NFAT1/NFATc2 [29], we first confirmed that the NFAT5 shRNAs inhibited only this factor, and not NFATc proteins. We tested both shRNAs with the same reporter, 9×NFAT-Luc, which can be activated independently by NFAT5 in response to hypertonic stress, or by the calcineurin-dependent NFATc proteins in response to PMA and ionomycin [25] (Fig. 4C). With regard to the cyclin A2 and B1 promoters, suppression of NFAT5 did not have a substantial effect on their activity after 8 and 24 hours of hypertonic stress (Fig. 4C). Although one of the shRNAs (shN5-1) slightly increased the activity of the cyclin A2 promoter after 24 hours in isotonic conditions, this effect was likely non-specific, since it was not observed with another shRNA (shN5-3) that was comparably effective in inhibiting NFAT5. On the other hand, overexpression of NFAT5 enhanced the activity of the control 9×NFAT-Luc reporter during both short (8 hours) and longer (24 hours) hypertonicity treatments, and did not affect the cyclin B1 promoter (Fig. 4D). We observed that overexpressed NFAT5 caused a transient increase in the activity of the cyclin A2 promoter at 8 hours (Fig. 4D). However, since this effect was moderate, occurred in both isotonic and hypertonic conditions, and we had not observed a decrease in cyclin A2 promoter activity upon suppessing NFAT5 (Fig. 4C), it is unclear whether it reflected a meaningful function of NFAT5. Altogether, these results suggested that NFAT5 was not required for the activity of cyclins A2 and B1 promoters under hypertonic stress.

### 4) Regulation of NFAT5 throughout the cell cycle

**Figure 5:**
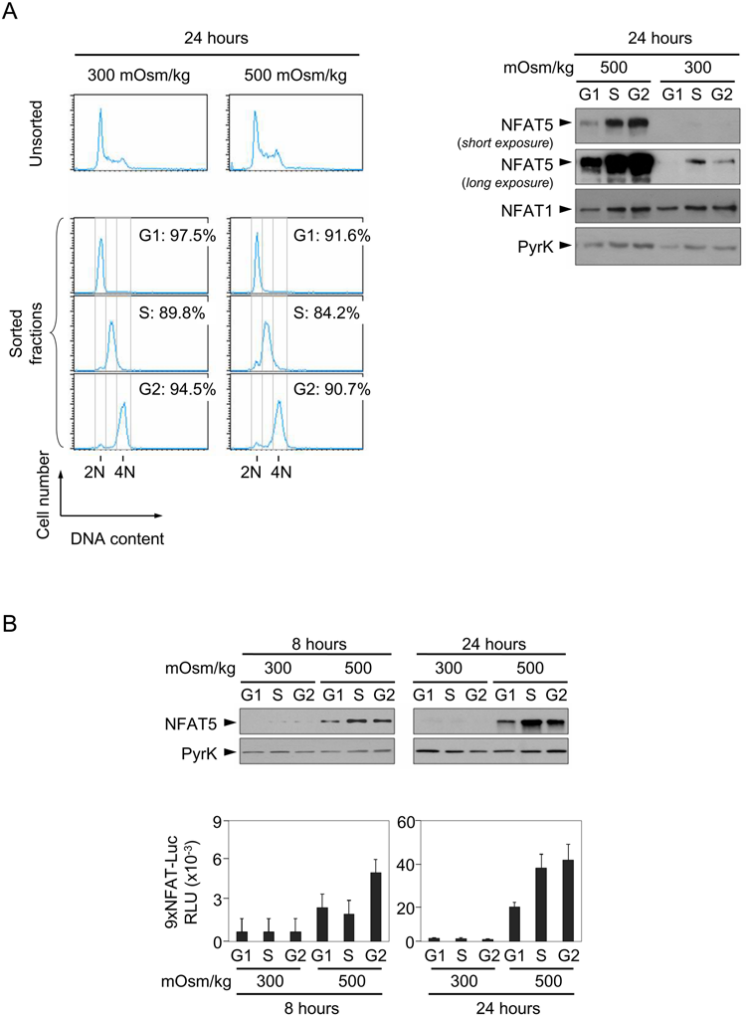
Expression and activity of NFAT5 throughout the cell cycle. A) Proliferating NFAT5^+/+^ T cells cultured during 24 hours in isotonic (300 mOsm/kg) or hypertonic (500 mOsm/kg) conditions were labeled with Hoechst 33342 and sorted according to DNA content as G0/G1, S or G2/M phase. Cell cycle histograms in the left panel show the efficiency of the sorting. Sorted cells were lysed and equal amounts of protein from each lysate were analyzed by Western blot with anti-NFAT5 antibody. NFAT1 and anti-pyruvate kinase (PyrK) are shown as protein loading controls. The result is representative of three independent experiments. B) Transgenic 9×NFAT-Luc T cells were labeled with Hoechst 33342 and sorted after 8 and 24 hours of exposure to hypertonicity. Sorted cells were split for Western blot and luciferase activity assays. A representative Western blot analysis (upper panel) is shown of three independently performed. Pyruvate kinase (PyrK) is shown as protein loading control. 9×NFAT-Luc reporter activity normalized to endogenous LDH is shown in the lower panel. Results are the mean±SEM of three independent experiments.

Since lack of NFAT5 caused defective cell cycle regulation in lymphocytes exposed to hypertonic stress, we wondered whether the activity of this factor varied in specific phases of the cell cycle. In order to measure the expression and activity of NFAT5 simultaneously, we used transgenic T cells with an integrated NFAT5-responsive reporter, 9×NFAT-Luc [25]. T cells proliferating under isotonic or hypertonic conditions were labeled with the DNA dye Hoechst 33342, and sorted by cell cycle phase. We observed that the expression of NFAT5 was already higher in S and G2/M in isotonic conditions, and increased considerably under osmotic stress, with a pronounced accumulation in both phases (Fig. 5A). With regard to its activity, we observed that after 8 hours of hypertonic stress, with the cell cycle still arrested, NFAT5 was active in all phases but had greater activity in G2/M. By 24 hours, when cells had resumed proliferation, NFAT5 activity increased substantially with respect to 8 hours and was highest in S and G2/M, which were the phases more severely affected by hypertonicity in NFAT5^−/−^ cells (Fig. 5B).

### 5) Defective induction of cell cycle regulators in response to T cell receptor stimulation in NFAT5^−/−^ lymphocytes exposed to pathologic hypertonicity

In the experiments shown above, proliferating lymphocytes were exposed to hypertonic conditions of 500 mOsm/kg, which have been routinely utilized throughout the literature to analyze NFAT5-dependent responses in diverse cell types. However, switching cell cultures from an isotonic medium at 300 mOsm/kg to 500 mOsm/kg constitutes a rather severe osmotic shock that might not reflect pathophysiological situations. Dehydration [30], as well as anisosmotic disorders described in patients [31]–[37], and deficiency of osmoregulatory proteins such as vasopressin V2 receptor [38], or aquaporins 1 and 2, in mouse models [39], [40], have been reported to cause hypernatremia with plasma osmolality values of 360–430 mOsm/kg. These conditions would expose different cell types, including lymphocytes, to a hypertonic milieu.

**Figure 6:**
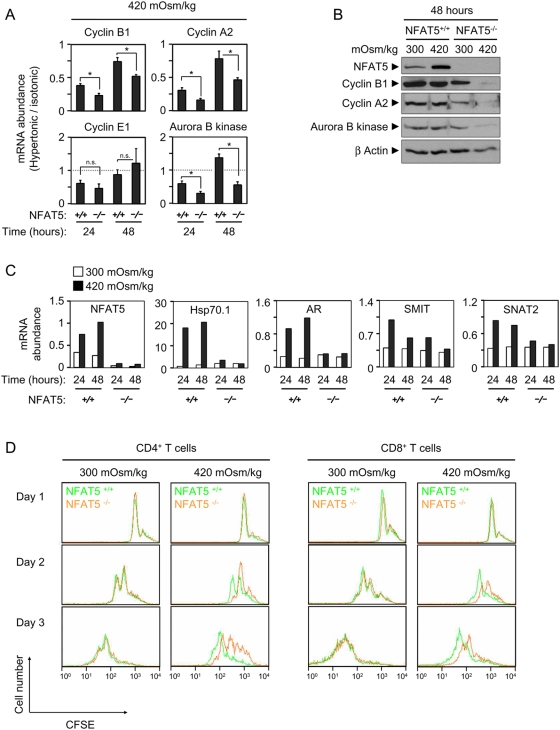
Effect of pathologic hypertonicity on the induction of cyclins and aurora B kinase in response to T cell receptor stimulation in NFAT5^−/−^ T cells. A) Splenocytes were induced to proliferate with anti-CD3/CD28 antibodies plus IL-2 in isotonic or moderately hypertonic medium. Cultures were harvested at the indicated time points and depleted of B cells. RNA was isolated and analyzed by RT-qPCR. All values were normalized to each respective L32 mRNA level. Graphs represent the mRNA abundance of the indicated gene products in hypertonic relative to isotonic conditions. Values correspond to the mean±SEM of three independent experiments (* = p<0.05; n.s. = not statistically significant). B) T cells were cultured and purified as in A), and the expression of the indicated proteins was analyzed by Western blot in cell lysates. β-actin was used as protein loading control. The experiment shown is representative of three independently performed (see also Fig. S6). C) T cells were cultured and purified as in A), and the abundance of the indicated mRNAs was analyzed by RT-qPCR. All values were normalized to each respective L32 mRNA level. A representative experiment of three independently performed is shown. D) Splenocytes labeled with CFSE were induced to proliferate with anti-CD3/CD28 antibodies plus IL-2 in isotonic (300 mOsm/kg) or moderately hypertonic medium (420 mOsm/kg). CFSE fluorescence in the CD4 and CD8 T cell subsets was analyzed by two-color flow cytometry in the population of live cells after 1, 2 and 3 days of culture. The experiment is representative of three independently performed.

We thus tested whether fresh NFAT5^−/−^ T cells exhibited defects in the expression of different cell cycle regulators when induced to proliferate in moderately hypertonic medium (380–420 mOsm/kg). Induction of cyclins A2, B1 and aurora B kinase by CD3/CD28 stimulation was more severely impaired in NFAT5^−/−^ T cells at 420 mOsm/kg than in wild-type ones (Figs. 6A and 6B). Cyclin E1, though, was comparably affected in both cell types. We also observed that aurora B kinase, which has been recently shown to regulate cell cycle progression in T cells [41], was downregulated in hypertonicity-treated NFAT5^−/−^ T cells (Figs. 6A and 6B). Similar results were obtained in cells stimulated with concanavalin A and IL-2 (Fig. S6). In the same experiments, NFAT5-deficient lymphocytes displayed a defective osmoprotective response, with poor induction of Hsp70.1, aldose reductase, SMIT, and SNAT2 (Fig. 6C). Altogether, these results showed that NFAT5 deficiency could impair the expression of major cell cycle regulators in primary cells exposed to pathologic hypertonic stress conditions. Finally, we assessed whether sustained moderate hypertonic stress affected the proliferative capacity of NFAT5-deficient lymphocytes. CFSE-labeled splenocytes were stimulated with anti-CD3/CD28 antibodies and IL-2 during 3 days in isotonic (300 mOsm/kg) or hypertonic media (420 mOsm/kg). In this assay, CFSE fluorescence decreases with the number of cell divisions throughout the culture. Analysis of CFSE fluorescence in viable T cells showed that both CD4 and CD8 NFAT5^−/−^ lymphocytes proliferated comparably to wild-type cells along a 3-day period of stimulation with anti CD3/CD28 antibodies and IL-2 in isotonic conditions, but exhibited a greater decrease in proliferative capacity upon exposure to hypertonic stress (Fig. 6D). This result indicated that the early defect in induction of cell cycle regulators in NFAT5^−/−^ T cells under hypertonic stress was followed by an impaired proliferative capacity at later time points.

## Discussion

One of our main findings, not addressed in previous studies, has been to draw a clear distinction between NFAT5-regulated and NFAT5-independent events in the osmotic stress response of proliferating mammalian cells. Our results show that this response developed in two phases. The early phase was NFAT5-independent and characterized by a sharp induction of p53 and other genotoxic stress markers (p21, GADD45α and β), downregulation of cyclins mRNA, and acute cell cycle arrest in S and G2/M. This initial response changed to an NFAT5-regulated adaptive phase in wild-type cells, which induced osmoprotective gene products such as Hsp70.1, SMIT, SNAT2 and TauT, resumed cyclin expression, and reactivated the cell cycle. In contrast, NFAT5^−/−^ cells showed poor induction of osmoprotective genes, manifested an extended early stress phase with prolonged accumulation of p53, and exhibited a greater viability loss than wild-type cells by 24 hours. Surviving NFAT5^−/−^ cells had downregulated stress markers but displayed a reduced DNA replication rate and defective cell cycle progression associated with the inability to maintain the expression of cyclins that regulate G1 to G2/M progression. The finding that S and G2/M phases were highly sensitive to hypertonic stress in NFAT5-deficient cells was consistent with the observation that NFAT5 expression and activity were enhanced in these phases in wild-type cells proliferating under sustained hypertonic conditions.

We observed defective expression of osmoprotective gene products and cell cycle regulators in actively proliferating NFAT5^−/−^ lymphocytes subjected to an osmotic shock of 500 mOsm/kg, as well as in fresh T cells that were induced to proliferate in moderately hypertonic media (380–420 mOsm/kg), in the range of osmolality levels described in plasma of dehydrated animals [30], patients with hypernatremic disorders [31]–[37] and mice deficient in osmoregulatory proteins [38]–[40]. Previous work by others and us had shown that tonicity levels of 380–420 mOsm/kg were sufficient to mobilize NFAT5 in neurons [42] and lymphocytes [25], and inhibited the proliferation of NFAT5-deficient lymphocytes [24]. Our findings, together with those by other authors, collectively indicate that the osmoprotective role NFAT5 is not limited to the renal medulla, but extends to different cell types which might be exposed to hypertonic stress under situations that affect the osmotic equilibrium of the organism.

We also found that the number and proportion of T cells were not altered *in vivo* in our conditional knockout mice (Fig. S2E). This is in contrast with previous findings from the Ho laboratory showing that mice expressing a dominant negative NFAT5 construct in thymocytes had reduced thymic cellularity and decreased proportions of T cells in spleen [18]. Later work by the same group showed a similar phenotype in mice in which one NFAT5 allele had been truncated and the expressed product was suggested to act as an endogenous dominant negative [24]. Nonetheless, lymphocytes from our conditional knockout mice and those described in [24] expanded normally in isotonic medium and showed a comparable impairment of their proliferative capacity when cultured under hypertonic conditions, indicating that NFAT5-deficient T cells in these mouse models react similarly to osmotic stress *in vitro*. The reasons for the different *in vivo* T cell phenotypes between mouse models are unclear. Go et al., proposed that the moderately hypertonic millieu (∼330 mOsm/kg) of the thymus and other lymphoid organs could impair the development and function of NFAT5-deficient lymphocytes [24], whereas our results indicate that these environments may not be unfavorable for mature NFAT5^−/−^ T cells *in vivo*, and suggest that this factor might play a more important role during abnormal hypertonic stress conditions. Another difference between these mouse models is that T cells in our conditional knockout mice were NFAT5-null, whereas thymocytes and T cells in the two previous models coexpressed functional NFAT5 molecules together with truncated dominant negative products, which might have additional effects on T cell development. On the other hand, a recent article described that mice with reduced expression of the guanine nucleotide exchange factor Brx had substantially decreased NFAT5 expression in spleen and renal medulla [43], exhibited B cell defects and reduced splenocyte counts, but had normal T cell proportions. These findings suggest that different NFAT5-deficient mouse models could exhibit variable degrees of immune dysfunction depending on whether NFAT5 is impaired only in mature T cells or in other cell types.

Our results indicate that lack of NFAT5 in cells exposed to hypertonicity could lead to different outcomes, with a proportion of the cells dying, probably as result of an enhanced genotoxic stress response, whereas surviving cells did not exhibit conspicuous symptoms of DNA damage but had a substantially reduced expression of cyclins and aurora B kinase. Previous work had shown that inhibition of NFAT5 could cause symptoms of DNA damage in non-proliferating cells [28], but until now it was not known that the abundance of specific cell cycle regulators was sensitive to hypertonicity and the lack of NFAT5. Defective expression of cyclins and aurora B kinase might underlie the cell cycle defects of NFAT5^−/−^ cells under hypertonic stress, since cyclins A2 and B1 are needed for S and G2/M progression in different cell types [44], [45], and aurora B kinase is a positive regulator of cell cycle progression through G1/S to M in T cells [41]. Here it is important to notice that lack of NFAT5 did not shut down all processes, and for instance NFAT5-deficient cells were able to reinduce cyclin D3 after its initial downregulation by hypertonic stress. Our results also indicate that expression of these cell cycle regulators was affected at different levels in NFAT5-deficient cells, as loss of cyclins A2, B1 and aurora B kinase correlated with a reduction in their mRNA abundance, whereas the decrease in cyclin E1 did not appear to be solely attributable to mRNA deficiency, and might be due to reduced protein stability and/or synthesis rate. On the other hand, suppression or overexpression of NFAT5 did not substantially affect the activity of cyclins A2 and B1 promoters under hypertonic stress. Although these results do not rule out that NFAT5 might have an effect on other regulatory regions of cyclin genes, they suggest that it does not control the activity of their promoters. In this regard, NFAT5 differs from the NFATc protein NFAT1/c2, which can repress the expression of cyclins E, A2 and B1 during T cell activation by antigen [46]. At least for cyclin A2, this repression was mediated by inhibition of the activity of its promoter by NFAT1/c2 [29]. In view of these observations, we propose that lack of NFAT5 might indirectly perturb the expression of cyclins and possibly other genes in cells exposed to osmotic stress as result of the combined deficiency in osmoprotective gene products, which are needed for the adaptation to hypertonic conditions and are the primary targets of NFAT5. This interpretation is consistent with the finding that cells lacking the chaperones Hsp70 and Hspa4l/Osp94, whose induction by hypertonicity is NFAT5-dependent, have defects similar to those of NFAT5^−/−^ cells: Hsp70-deficient fibroblasts and renal medullary cells have reduced viability under hypertonic conditions [47], and Hspa4l/Osp94-deficient mice exhibited loss of renal medullary cells similarly to NFAT5-deficient mice [20], [48].

Here we have used primary lymphocytes as a cell model to study the response of proliferating NFAT5-deficient cells to pathologic hypertonic stress conditions. We have shown that moderate osmotic stress induced an NFAT5-dependent osmoprotective gene expression program in T cells, and that lack of this factor led to defects in the expression of several cyclins and aurora B kinase. The feasibility of generating mice lacking NFAT5 in specific cell lineages will be useful to determine the sensitivity of other types of primary cells to pathophysiologic alterations of tonicity, and to elucidate the role of this factor in different cellular functions and processes.

## Materials and Methods

### Generation of NFAT5 conditional mice

A targeting vector was designed to flank exon 6 of the mouse *NFAT5* gene with two *loxP* sites (Fig. S1). A BamHI fragment of the mouse *NFAT5* genomic locus isolated from a P1 clone was used. A 3.3-kb ApaLI-AvrII fragment was used as 5′ homology region, and a 4.8-kb EcoRI-XbaI fragment was used as 3′ homology region. One *loxP* site was introduced 5′ to exon 6, in the 1.8-kb AvrII-EcoRI fragment. An *frt* site-flanked selection cassette, with a neomycin resistance gene, the Flpe cDNA cloned under control of the ACE promoter [49], and the second *loxP* site, was inserted into an EcoRI site in the sixth intron of the *NFAT5* gene. The targeting vector also contained a thymidine kinase gene was used for negative selection of clones with random integration of the targeting vector.

Bruce-4 embryonic stem (ES) cells [50] derived from C57BL/6 mice were transfected, cultured, and selected as previously described [20]. Of 800 G418 (neomycin) and gancyclovir-resistant colonies, 3 were identified as homologous recombinants with cointegration of the second *loxP* site by Southern blot analysis of BamHI-digested DNA, using a probe spanning the exon 5 as 5′ external probe (Fig. S1) and Neo as a 3′ probe. ES clones with the appropriately targeted allele were injected into BALB/c blastocysts to generate chimeric mice, which transmitted the targeted allele to their progeny. All mice were maintained on a pure C57BL/6 genetic background. The *frt*-flanked neomycin resistance cassette was removed through intercrossing with FLPe-deleter mice [51]. Mice lacking NFAT5 in T cells were obtained after successive crosses of NFAT5^Flox/Flox^ mice with CD4-Cre transgenic mice, in which the Cre recombinase is under the control of the mouse CD4 promoter/enhancer/silencer [52]. In these mice, Cre is induced during the double positive stage of thymocyte development after cells have rearranged the T cell receptor. Mice were bred and maintained in specific pathogen-free conditions, and animal handling was performed according to institutional guidelines approved by the ethical committee (PRBB Animal Care and Use Committee). The CD4-Cre transgenic mouse strain [52] was obtained from the Jackson Laboratory (Bar Harbor, ME).

### 9×NFAT-Luc mice and p53^−/−^ mice

9×NFAT-Luc mice (line 15.1) in FVB background were previously described [25], [53]. p53^−/−^ mice were obtained from the Jackson Laboratory and have been previously described [54].

### Lymphocytes

Primary mouse T cells were obtained from spleens of CD4-Cre^+^/NFAT5^Flox/Flox^ mice (hereafter abbreviated as NFAT5^−/−^), littermate CD4-Cre^−^/NFAT5^Flox/Flox^ mice (wild-type), 9×NFAT-Luc mice, p53^−/−^ and p53^+/+^ mice of 8–12 weeks of age. We confirmed that NFAT5-expressing T cells derived from CD4-Cre^−^/NFAT5^Flox/Flox^ or CD4-Cre^+^/NFAT5^+/+^ mice were indistinguishable in their response to the stresses tested (not shown). Splenocytes were isolated by density gradient centrifugation with Lymphoprep™ (Axis-Shield PoC AS, Oslo, Norway). Proliferating T cells were obtained by activating splenocytes (2.5×10^6^ cells/ml) with 2.5 µg/ml concanavalin A (Cat. C-2010, SIGMA) plus 25 ng/ml recombinant human IL-2 (Proleukin; Chiron, formerly Eurocetus. Amsterdam, The Netherlands) in culture medium (Dulbecco's Modified Eagle Medium DMEM, Gibco. Paisley, UK), supplemented with 10% fetal bovine serum (Cat. CH30160.03 Hyclone, Logan, UT, USA), non-essential amino acids (Gibco), 2 mM L-glutamine (Gibco), 50 µM beta-mercaptoethanol (Gibco), 1 mM sodium pyruvate (Gibco) and antibiotics penicillin and streptomycin (Gibco). Splenocytes cultures grown in this medium for 72 hours were then cleaned of dead cells and debris by centrifugation on Lymphoprep™, washed and replated in fresh medium supplemented with IL-2 for an additional 24 hours, after which both wild-type and NFAT5-conditional knockout cultures had >95% CD3^+^, TCRβ^+^ T cells (not shown). Before subjecting T cells to hypertonic conditions, dead cells and debris in the cultures were again removed by centrifugation on a Lymphoprep™ cushion. Then, T cells were adjusted to 0.5×10^6^ cells/ml in medium supplemented with 25 ng/ml IL-2, and cultured under isotonic or hypertonic conditions as indicated in the figure legends. Proliferating B cells were obtained by culturing splenocytes (2×10^6^ cells/ml) with 25 µg/ml of lipopolysaccharide (LPS, Cat. L7261, SIGMA) during 7 days. For Figs. 6 and S6, fresh splenocytes were induced to proliferate with hamster anti-mouse CD3 (1 µg/10^6^ T cells) plus hamster anti-mouse CD28 (1 µg/10^6^ T cells) antibodies and seeded onto goat-anti hamster IgG coated plates (0.6 µg/cm^2^), or stimulated with concanavalin A and IL-2 as described above. Cells were either grown in isotonic or moderately hypertonic media as indicated in figure legends. Before lysing cells for protein and mRNA analysis, samples were depleted of remaining B cells by incubation with sheep anti-mouse IgG magnetic beads (Dynabeads Cat. 110.31. Dynal Biotech, Invitrogen. Paisley, UK).

### Hypertonic stress

The osmolality of the culture medium was measured in a Fiske ONE TEN osmometer (Fiske Associates. Norwood, MA, USA) or with a VAPRO 5520 vapor pressure osmometer (Wescor. Logan, UT, USA). Since the T cell medium with supplements had an osmolality of 330 mOsm/kg, we adjusted it to an isotonic baseline of 300 mOsm/kg by adding 10% sterile H_2_O (Milli-Q Biocel A10. Millipore, Bedford, MA, USA). This medium was made hypertonic by adding NaCl from a sterile 4 M stock solution. Over an isotonic baseline of 300 mOsm/kg, addition of 40 mM NaCl raised the osmolarity to 380 mOsm/kg, 60 mM NaCl to 420 mOsm/kg, and 100 mM NaCl to 500 mOsm/kg.

### Antibodies

The anti-NFAT5 polyclonal antibody (Cat. PA1-023) was from Affinity Bioreagents (Golden, CO, USA) and recognizes a carboxy-terminal epitope (DLLVSLQNQGNNLTGSF). The anti-NFAT5 polyclonal antibody recognizing the N-terminal region of NFAT5 was previously described [7]. Rabbit polyclonal anti-phospho-p53 (Ser15) (Cat. 9284), mouse monoclonal anti-p53 (Cat. 2524) and mouse monoclonal anti-cyclin D3 (Cat. 2936) were from Cell Signaling Technology (Danvers, MA, USA); mouse monoclonal anti-phospho-histone H2AX (Ser139, γH2AX) (Cat. 05-636) was purchased from Upstate Technologies (Lake Placid, NY, USA); mouse monoclonal anti-BrdU antibody (Cat. 555627) was purchased from BD Pharmingen (San Diego, CA, USA). The anti-NFAT1 antibody (anti-NFAT1-C) has been described [55]. Anti-CD3-PE (Cat. 553064), anti-Thy1.2-PE (Cat. 553090) and anti-B220-PE (Cat. 553006) were from BD Biosciences. Anti-cyclin A2 (Cat. sc-751), anti-cyclin B1 (Cat. sc-245), anti-cyclin E1 (Cat. sc-481) and anti p21 (Cat. sc-397) were from Santa Cruz Biotechnology (Santa Cruz, CA, USA). Anti-aurora B kinase (Cat. ab2254) was from Abcam (Cambridge, UK). Hamster anti-mouse CD3 (Cat. 553058) and hamster anti-mouse CD28 (Cat. 553295) were from BD Biosciences; goat anti-hamster IgG (Cat. 55397) was from MP Biomedicals (Illkirch, France). Goat anti-pyruvate kinase (AB1235) was purchased from Chemicon (Hampshire, UK). Anti-β-actin (Cat. A5441) was from SIGMA (Steinheim, Germany). FITC-labeled anti-mouse IgG (Cat. F0313), FITC-labeled anti-rabbit IgG (Cat. F0054), and HRP-labeled anti-goat IgG (Cat. P010.60) were from DAKO (Glostrup, Denmark). HRP-labeled anti–mouse IgG (Cat. NA931V), and HRP-labeled anti-rabbit IgG (Cat. NA934V) were from Amersham (Buckinghamshire, UK).

### Surface marker analysis

2×10^5^ cells were blocked for 20 minutes in 1× PBS containing 3% fetal calf serum (FCS), 0.1% sodium azide, and anti-Fcγ receptor antibody (1 µg/10^6^ cells) (BD Biosciences, Cat. 553142). Cells were then incubated with surface marker-specific antibodies in the same solution (1 µg/10^6^ cells) and analyzed with a FACScan flow cytometer (BD Biosciences) and Cellquest software.

### Viability, apoptosis, and cell cycle analysis

Flow cytometry was done with a BD LSR flow cytometer (BD Biosciences). For viability and cell cycle analysis, cells were labeled during the last hour of culture with the DNA dye Hoechst 33342 (SIGMA) (5 µg/ml, 60 minutes in incubator at 37°C with 5% CO_2_). For the determination of apoptosis, cells were first labeled with Hoechst 33342 and then stained with annexin-V Fluos (Roche. Manheim, Germany) during 30 minutes on ice. Viability was determined by flow cytometry analysis of forward and side scatter parameters (FSC/SSC) together with DNA content. Non-viable cells had a DNA content <2N and were readily identified by their distinct position in the FSC/SSC plots (Fig. 3B). Cell proliferation was also analyzed in CFSE-labeling experiments. Briefly, splenocytes were labeled with 5 µM carboxyfluorescein diacetate succinimidyl ester (CFSE, Molecular Probes, Invitrogen) at day 0 and then stimulated with anti-CD3/CD28 antibodies plus IL-2 during 72 hours in isotonic or hypertonic conditions. The decrease in CFSE fluorescence intensity in CD4 and CD8 T cells, which was proportional to the number of cell divisions, was analyzed by two-color flow cytometry in the population of live cells.

### Determination of DNA replication by BrdU incorporation

Cells were pulse-labeled during 30 min (in a 37°C, 5% CO_2_ incubator) with 10 µM BrdU (Cat. B5002, SIGMA) and then fixed in 70% ethanol. BrdU was detected with a monoclonal mouse anti-BrdU antibody after acidic denaturation following the protocol supplied by the manufacturer (BD Pharmingen). Labeled cells were then stained with propidium iodide in RNase A-containing solution to simultaneously analyze DNA replication and cell cycle.

### Intracellular detection of γH2AX and phospho-p53 (Ser-15)

Cells were labeled following an intracellular staining protocol previously described [56]. Briefly, cells were fixed in 1.5% paraformaldehyde (SIGMA) on ice for 15 minutes, and permeabilized with 70% ethanol at −20°C for at least 2 hours. Ethanol was removed by centrifugation and two washes with PBS, and cells were incubated with mouse monoclonal anti-γH2AX antibody (1 µg/10^6^ cells) or rabbit polyclonal anti-phospho-p53 (Ser15) (1 µg/10^6^ cells) for 2 hours. Bound primary antibodies were detected by incubating cells with FITC-labeled secondary antibodies for 1 hour at room temperature and protected from light. DNA was stained with 5 µg/ml Hoechst 33342 for 30 minutes at room temperature.

### Comet assay

Alkaline comet assay to visualize DNA damage was done using the Trevigen kit (catalog 4250-050-K), according to the manufacturer's instructions (Trevigen, Gaithersburg, MD, USA). In order to assess DNA breaks only in the population of live cells, dead cells were removed by centrifugation on a Lymphoprep™ cushion and excluded from the assay.

### Cell sorting

Cells were labeled with Hoechst 33342 as indicated above, sorted with a FACSvantage flow cytometry system (BD Biosciences) according to their cell cycle phase; G0/G1, S, or G2/M, collected at 4°C and lysed immediately after sorting. The efficiency of the sorting was routinely verified by analyzing the cell cycle distribution of the sorted fractions.

### Protein sample preparation and Western blot Analysis

Cells were lysed (30 minutes at 4°C) in 50 mM HEPES (pH 7.4), 80 mM NaCl, 5 mM MgCl_2_, 10 mM EDTA, 5 mM sodium pyrophosphate, 1% Triton X-100, 20 mM β-glycerophosphate, and protease inhibitors PMSF, leupeptin (SIGMA), aprotinin (Roche), and pepstatin A (SIGMA). Lysates were cleared by centrifugation (15,000g, 15 minutes, 4°C) and the protein concentration in the supernatants was determined using the BCA Protein Assay (Pierce, Rockford, IL, USA). Equal amounts of protein from each sample were separated in SDS-polyacrylamide gels under reducing conditions, transferred to PVDF membranes (Immobilon-P. Millipore, Bedford, MA, USA), and detected with specific primary antibodies followed by HRP-labeled secondary antibodies and enhanced chemiluminescence (Supersignal West Pico Chemiluminescent Substrate, Pierce). Pyruvate kinase or β-actin were used as protein loading controls.

### Real-time quantitative PCR (RT-qPCR)

Total RNA was isolated using the RNeasy kit (Cat. 74104. Quiagen. Qiagen Iberia S.L., Madrid, Spain) following manufacturer's instructions. 2–3 µg of total RNA was retro-transcribed to cDNA using SuperScript III reverse transcriptase and random primers (Invitrogen). For real-time quantitative PCR (RT-qPCR), Power SYBR Green PCR master mix (Applied Biosystems, Cat. 4367659) and an ABI7900HT sequence detection system (Applied Biosystems) were used following the manufacturer's instructions. Samples were normalized to L32 mRNA levels using the ABI Prism SDS 2.1 software. Primers were: mouse NFAT5: 5′-CAG CCA AAA GGG AAC TGG AG-3′ (Forward) and 5′-GAA AGC CTT GCT GTG TTC TG-3′ (Reverse); mouse L32: 5′-ACC AGT CAG ACC GAT ATG TG-3′ (Forward) and 5′-ATT GTG GAC CAG GAA CTT GC- 3′ (Reverse); mouse Hsp70.1: 5′-CTT CTA CAC ATC CAT CAC GC-3′ (Forward) and 5′-TTG AAG AAG TCC TGC AGC AG- 3′ (Reverse); mouse SMIT: 5′-ATG GTT GTC ATC AGC ATA GCA TGG-3′ (Forward) and 5′-GGT GGT GTG AGA AGA CTA ACA ATC-3′ (Reverse); mouse TauT: 5′-TAC TAT GCA GCT AGT GGT GTA TGC-3′ (Forward) and 5′-ACC TGG TCC TAT GAG AAT CTA ACG-3′ (Reverse); mouse aldose reductase (AR): 5′ TGA GCT GTG CCA AAC ACA AG-3′ (Forward) and 5′-GGA AGA AAC ACC TTG GCT AC-3′ (Reverse); mouse sodium-dependent neutral aminoacid transporter 2 (*Slc38a2*, SNAT2): 5′-GGC AAG GTA TGT CTG CCA TT (forward) and 5′-GGG TTT CAT CTT GGG ACA GA (reverse); mouse cyclin E1: 5′-CTG GAC TCT TCA CAC AGA TG-3′ (Forward) and 5′-CAT CCA CAC TTG CTC ACA AC- 3′ (Reverse); mouse cyclin A2: 5′-GAC CAA GAG AAT GTC AAC CC-3′ (Forward) and 5′-CAT CGT TTA TAG GAA GGT CC- 3′ (Reverse); mouse cyclin B1: 5′-AGT TAC TGC TGC TTC CAA GC-3′ (Forward) and 5′-GGT AGG GCT TTA ACA GTA CC- 3′ (Reverse); mouse aurora B kinase: 5′-AAG AGT CGG ACC TTC GAT GA-3′ (Forward) and 5′-CTC CCT GCA GAC CTA ACA GC- 3′ (Reverse); mouse GADD45α: 5′-AGA AGA CCG AAA GGA TGG AC-3′ (Forward) and 5′-GAT GTT GAT GTC GTT CTC GC- 3′ (Reverse); mouse GADD45β: 5′-CTG CTG CGA CAA TGA CAT TG-3′ (Forward) and 5′-GAC CCA TTG GTT ATT GCC TC- 3′ (Reverse); mouse GADD45γ: 5′-TGT TCG TGG ATC GCA CAA TG-3′ (Forward) and 5′-CTC ATC TTC TTC ATC GGC AG- 3′ (Reverse).

### DNA constructs

The luciferase reporter 9×NFAT-Luc was previously described [53]. Cyclin A2-862-Luc was kindly provided by Dr. J.B.P Viola (Division of Cellular Biology, National Cancer Institute (INCA); Rio de Janeiro, Brazil) and has been described [29]. Cyclin B1 reporter plasmid was kindly provided by Dr. A. Gewirtz (University of Pennsylvania, Philadelphia, USA) and has been described [57]. The transfection control plasmid TK-Renilla was from Promega (Promega Biotech Ibérica, Madrid, Spain). The GFP-specific shRNA in the pBSU6 vector was previously described [58], and the two NFAT5-specific shRNAs were done by inserting the following 21-nucleotide sequences complementary to NFAT5 mRNA in pBSU6: shNFAT5-1 (shN5-1), 5′-GGT CAA ACG ACG AGA TTG TGA-3′; and shNFAT5-3 (shN5-3), 5′-GGT CGA GCT GCG ATG CCC TCG-3′. The CMV-HA vector was from Clontech (Clontech Palo Alto, CA). Full-length human NFAT5, corresponding to human isoform NFAT5a (GenBank AF134870) tagged with 6 copies of Myc in its amino-terminal and the enhanced green fluorescence protein (GFP) at its carboxy-terminus (Myc-NFAT5-GFP) has been described [7].

### Transfections and reporter assays

The human T cell line Jurkat (Clone E6-1, American Type Culture Collection, #TIB 152) was kindly provided by Dr. J. Luban (Columbia Universtity College of Physicians and Surgeons, New York, NY) and maintained in Dulbecco's modified Eagle's Medium (DMEM) supplemented with 10% heat-inactivated fetal bovine serum, 2 mM L-glutamine, 1 mM sodium pyruvate and 50 µM beta-mercaptoethanol (Gibco. Pasley, UK). Cells (20×10^6^ cells/400 µl serum-free DMEM) were transfected by electroporation (260 V, 950 µF, with a Bio-Rad Gene Pulser. Bio-Rad, Hemel Hampstead, UK) with luciferase reporter plasmids (60 ng/10^6^ cells), TK-Renilla (0.1 µg/10^6^ cells) and shRNA vectors (1.8 µg/10^6^ cells) as indicated in figure legends. 36 hours post-transfection, cells were placed in fresh isotonic medium (300 mOsm/kg) and subjected to hypertonic conditions (500 mOsm/kg) or stimulated with 20 nM phorbol 12-myristate 13-acetate (PMA) plus 1 µM ionomycin (Calbiochem. Darmstadt, Germany) as indicated in figure legends. Luciferase and Renilla were measured with the Dual-luciferase reporter system (Promega) with a Berthold FB12 luminometer (Berthold, Pforzheim, Germany). Luciferase activity was normalized to Renilla. Luciferase activity in transgenic 9×NFAT-Luc lymphocytes was normalized to endogenous lactate dehydrogenase (LDH), in the same lysate. LDH was proportional to the number of viable cells and was measured with the CytoTox 96 Non-Radioactive Cytotoxicity Assay (Promega) [25].

### Statistical Analysis

Mean, standard error of the mean (SEM) and statistical significance (t-Student test) were calculated using Microsoft Excel software.

## Supporting Information

Figure S1 Generation of NFAT5-conditional knockout mice. A) Schematic representation of the targeting construct, in which exon 6, encoding the DNA binding loop in the DNA binding domain of NFAT5, was flanked by *loxP* sites. The vector contained an *frt*-flanked neomycin-resistance cassette (Neo) inserted at the EcoRI site downstream of exon 6 and upstream of the 3′ *loxP* site. Restriction sites in brackets indicate that they were inactivated during subcloning. Mouse ES clones with the correctly recombined allele were used to generate mice that were crossed to FLPe-deleter mice to produce NFAT5-floxed mice, without the Neo cassette, and with Exon 6 flanked by *loxP* sites so that it could be removed by the Cre recombinase.
(1.86 MB TIF)

Figure S2 Lack of NFAT5 expression in T lymphocytes from NFAT5-conditional knockout mice. A) Southern blot of genomic DNA extracted from T cells of wild-type (WT), NFAT5-floxed (Flox) mice, and mice obtained after crossing them with CD4-Cre transgenic mice (CD4-Cre^+^). Genomic DNA was digested with BamHI and hybridized to a probe for exon 5. B) Specific deletion of NFAT5 in T cells, but not in B cells of CD4-Cre^+^/NFAT5^Flox/Flox^ mice was confirmed by Western blotting with an antibody against a carboxy (C)-terminal epitope. The non-specific crossreacting band (n.s.) above NFAT5 serves as a loading control. C) Western blot detecting NFAT5 in activated T cells was performed with two different antibodies, specific for a C-terminal epitope and the amino (N)-terminal region respectively. The majority of T cells obtained from CD4-Cre^+^/NFAT5^Flox/Flox^ mice lacked NFAT5, although in some experiments we could detect a small proportion of cells (below 10%) that had escaped deletion. D) NFAT5 mRNA was analyzed by RT-qPCR (bars represent the mean±SEM of five independent experiments). E) Weight of mice and spleens, and splenocyte count after Lymphoprep TM gradient separation (n = 8, bars are the mean±SEM). Expression of surface markers Thy1.2, B220 and CD3 in fresh splenocytes was analyzed by flow cytometry (n = 5, values are the mean±SEM).
(2.20 MB TIF)

Figure S3 Cell cycle profile of proliferating NFAT5−/− T cells under hypertonic stress. NFAT5^+/+^ and NFAT5^−/−^ proliferating T cells were either maintained in isotonic conditions (300 mOsm/kg) or switched to hypertonic medium (500 mOsm/kg) for 8 and 24 hours. The upper panel shows DNA content histograms representing the cell cycle distribution in live cells: G0/G1, early S (ES), late S (LS), and G2/M. The lower panel, shows the cell cycle distribution in wild-type and NFAT5^−/−^ cells after 8 and 24 hours in isotonic or hypertonic conditions. Values are the mean±SEM of five independent experiments (* = p<0.05).
(2.15 MB TIF)

Figure S4 Induction of p53 and p21 in NFAT5−/− T cells in response to hypertonicity. A) Phospho-p53 (Ser15) was detected by intracellular staining in NFAT5^+/+^ and NFAT5^−/−^ T cells cultured in isotonic or hypertonic medium during 24 hours. Results correspond to cells gated as alive. Dot plots show one representative experiment. Bars on the right represent the mean±SEM of four independent experiments (* = p<0.05). B) Time course of p53-Ser15 phosphorylation, accumulation of total p53 and p21 in NFAT5^+/+^ and NFAT5^−/−^ T cells in response to hypertonicity were analyzed by Western blot. Pyruvate kinase (PyrK) is shown as protein loading control. C) Time course of NFAT5 and p21 induction in p53^+/+^ and p53^−/−^ T cells in response to hypertonicity. The experiment shown is representative of three independently performed.
(1.90 MB TIF)

Figure S5 Expression of cyclins in proliferating NFAT5−/− T cells upon exposure to hypertonic conditions. Expression of cyclins D3, E1, A2 and B1 was analyzed by Western blot in lysates of proliferating NFAT5^+/+^ and NFAT5^−/−^ T cells after 8 and 24 hours of hypertonicity treatment. Pyruvate kinase (PyrK) is shown as protein loading control.
(1.81 MB TIF)

Figure S6 Effect of hypertonicity on cyclin induction by mitogens or T cell receptor activation in NFAT5−/− T cells. Expression of cyclins A2 and B1 was analyzed by Western blot in lysates of NFAT5^+/+^ and NFAT5^−/−^ T lymphocytes induced to proliferate with (A) anti-CD3/CD28 antibodies plus IL-2 or (B) concanavalin A (ConA) plus IL-2 in isotonic or moderately hypertonic media. Pyruvate kinase (PyrK) was used as protein loading control.
(2.20 MB TIF)
