# Supplementary figures and images for: The Transcription Factor NFAT5 Is Required for Cyclin Expression and Cell Cycle Progression in Cells Exposed to Hypertonic Stress
Source: PLoS One. 2009 Apr 21;4(4):e5245. doi: 10.1371/journal.pone.0005245 (PMC2667631; doi:10.1371/journal.pone.0005245)

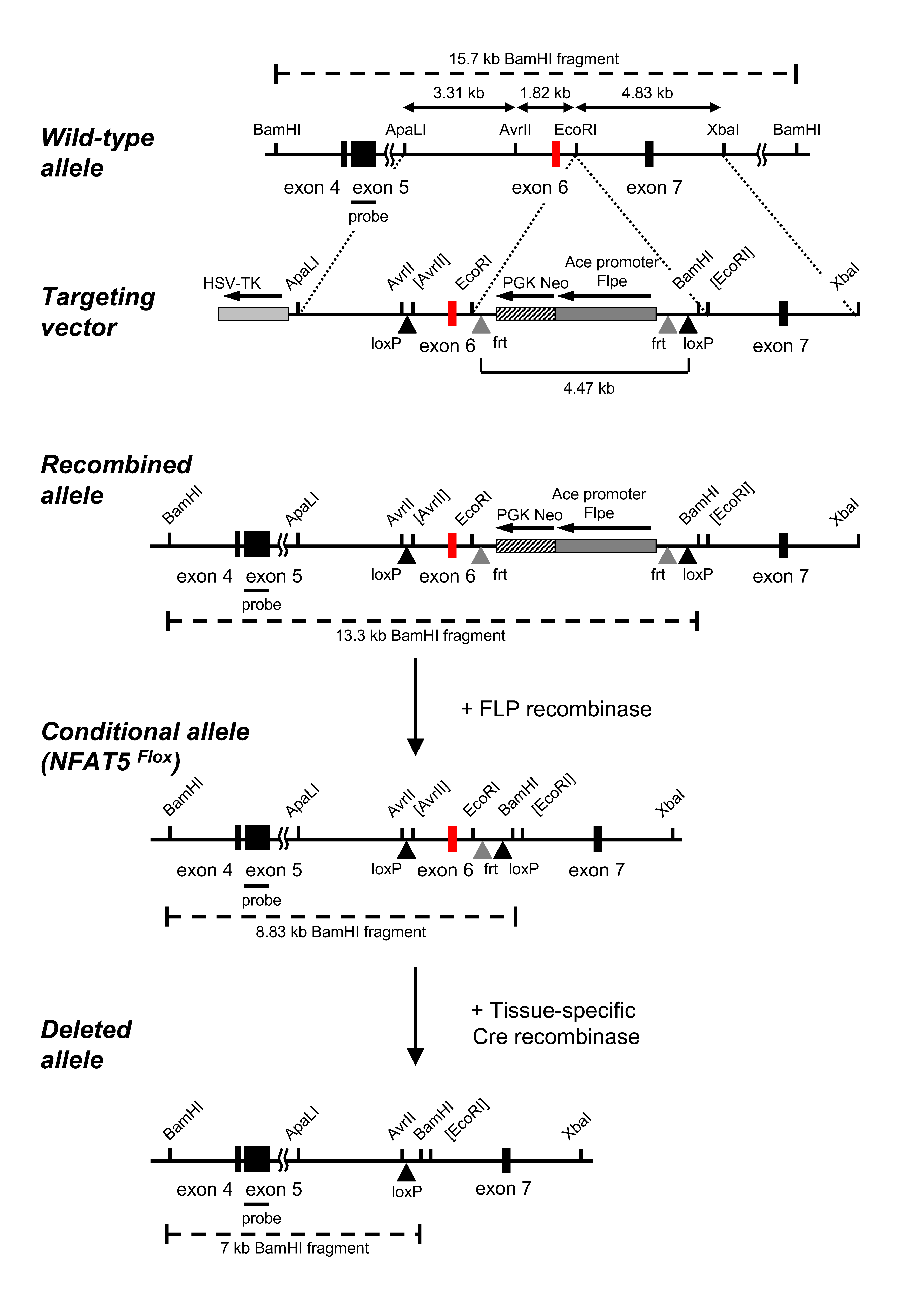

Supplement: Figure S1 — Generation of NFAT5-conditional knockout mice. A) Schematic representation of the targeting construct, in which exon 6, encoding the DNA binding loop in the DNA binding domain of NFAT5, was flanked by loxP sites. The vector contained an frt-flanked neomycin-resistance cassette (Neo) inserted at the EcoRI site downstream of exon 6 and upstream of the 3′ loxP site. Restriction sites in brackets indicate that they were inactivated during subcloning. Mouse ES clones with the correctly recombined allele were used to generate mice that were crossed to FLPe-deleter mice to produce NFAT5-floxed mice, without the Neo cassette, and with Exon 6 flanked by loxP sites so that it could be removed by the Cre recombinase. (1.86 MB TIF) [file pone.0005245.s001.tif]

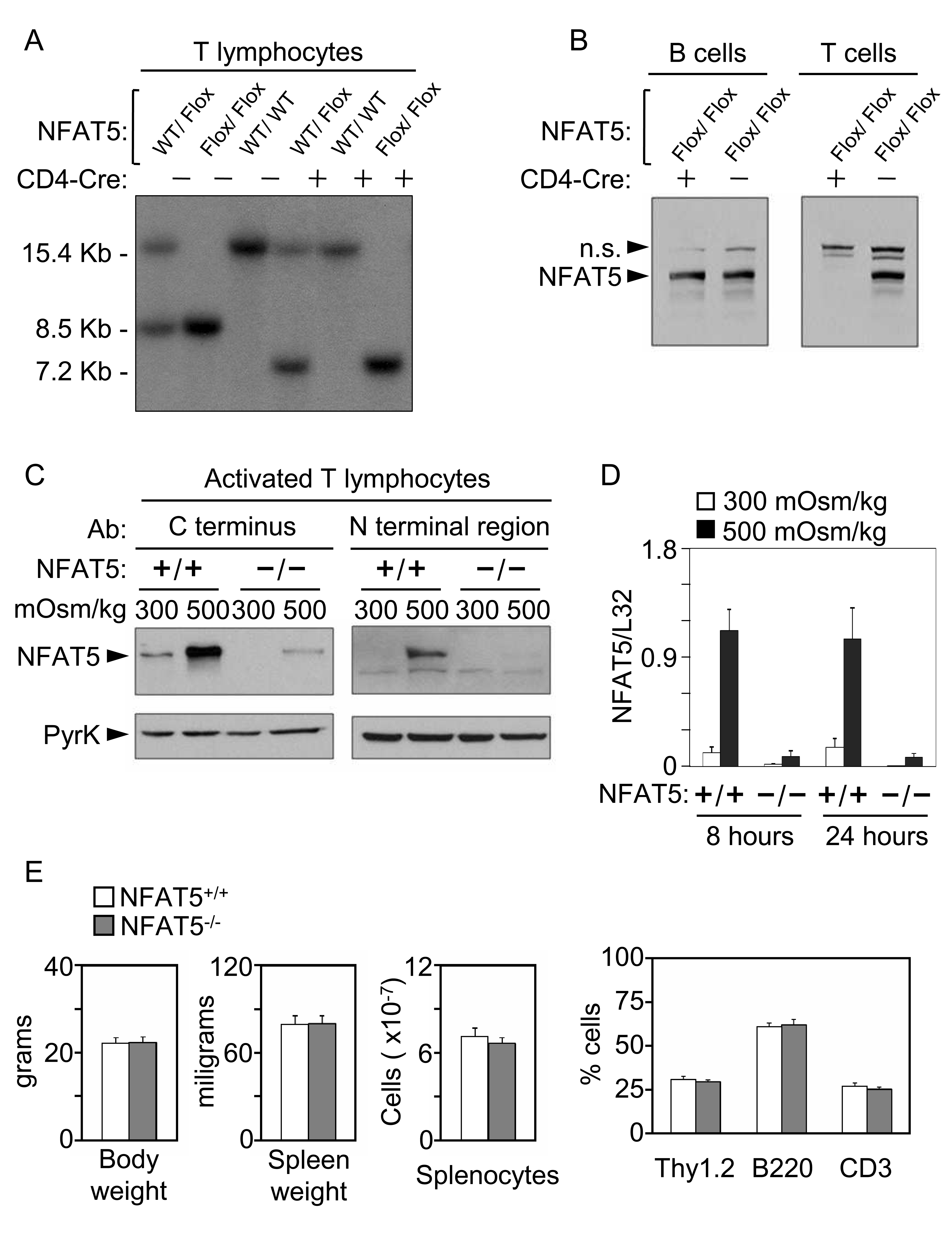

Supplement: Figure S2 — Lack of NFAT5 expression in T lymphocytes from NFAT5-conditional knockout mice. A) Southern blot of genomic DNA extracted from T cells of wild-type (WT), NFAT5-floxed (Flox) mice, and mice obtained after crossing them with CD4-Cre transgenic mice (CD4-Cre+). Genomic DNA was digested with BamHI and hybridized to a probe for exon 5. B) Specific deletion of NFAT5 in T cells, but not in B cells of CD4-Cre+/NFAT5Flox/Flox mice was confirmed by Western blotting with an antibody against a carboxy (C)-terminal epitope. The non-specific crossreacting band (n.s.) above NFAT5 serves as a loading control. C) Western blot detecting NFAT5 in activated T cells was performed with two different antibodies, specific for a C-terminal epitope and the amino (N)-terminal region respectively. The majority of T cells obtained from CD4-Cre+/NFAT5Flox/Flox mice lacked NFAT5, although in some experiments we could detect a small proportion of cells (below 10%) that had escaped deletion. D) NFAT5 mRNA was analyzed by RT-qPCR (bars represent the mean±SEM of five independent experiments). E) Weight of mice and spleens, and splenocyte count after Lymphoprep TM gradient separation (n = 8, bars are the mean±SEM). Expression of surface markers Thy1.2, B220 and CD3 in fresh splenocytes was analyzed by flow cytometry (n = 5, values are the mean±SEM). (2.20 MB TIF) [file pone.0005245.s002.tif]

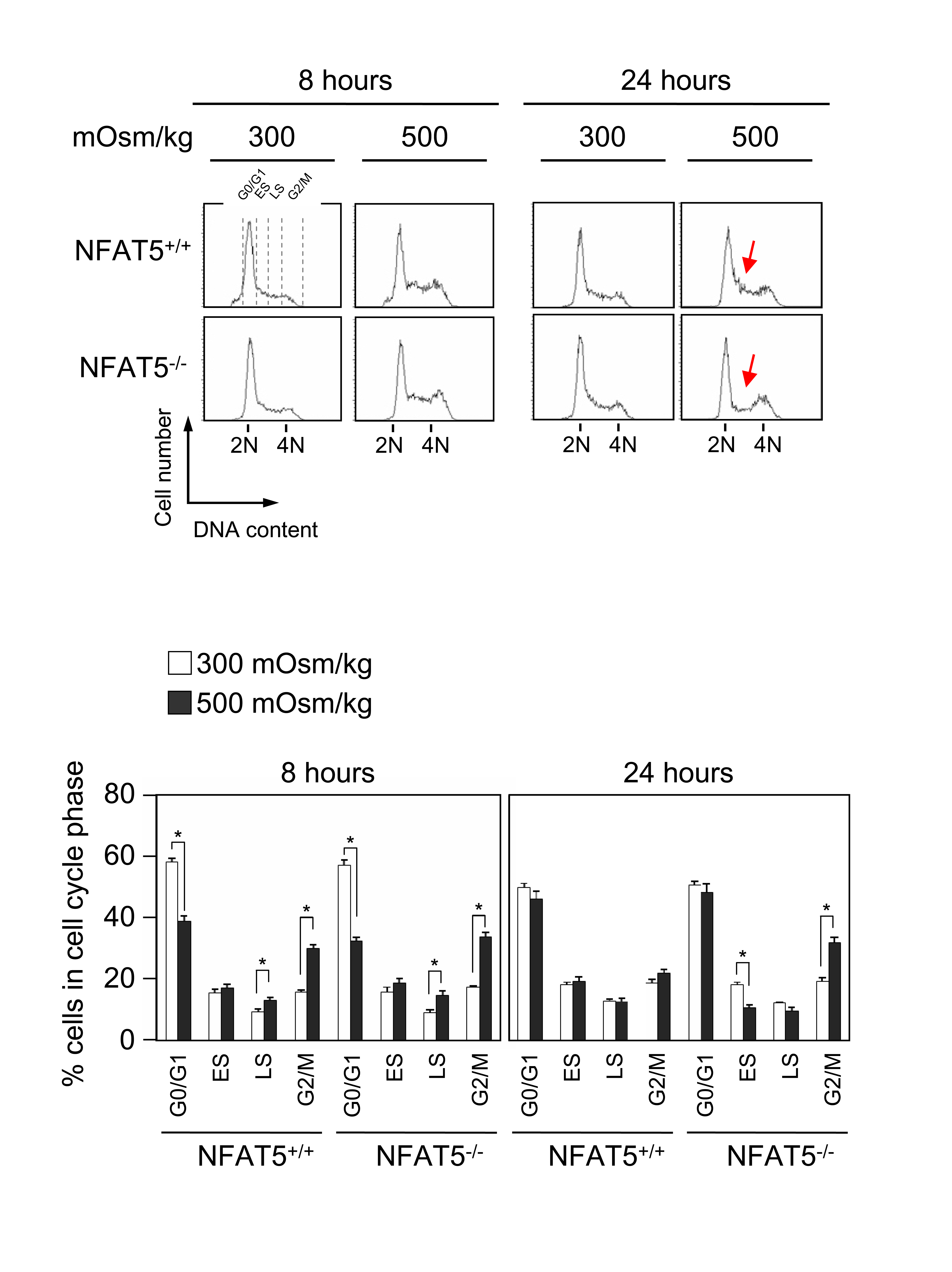

Supplement: Figure S3 — Cell cycle profile of proliferating NFAT5−/− T cells under hypertonic stress. NFAT5+/+ and NFAT5−/− proliferating T cells were either maintained in isotonic conditions (300 mOsm/kg) or switched to hypertonic medium (500 mOsm/kg) for 8 and 24 hours. The upper panel shows DNA content histograms representing the cell cycle distribution in live cells: G0/G1, early S (ES), late S (LS), and G2/M. The lower panel, shows the cell cycle distribution in wild-type and NFAT5−/− cells after 8 and 24 hours in isotonic or hypertonic conditions. Values are the mean±SEM of five independent experiments (* = p<0.05). (2.15 MB TIF) [file pone.0005245.s003.tif]

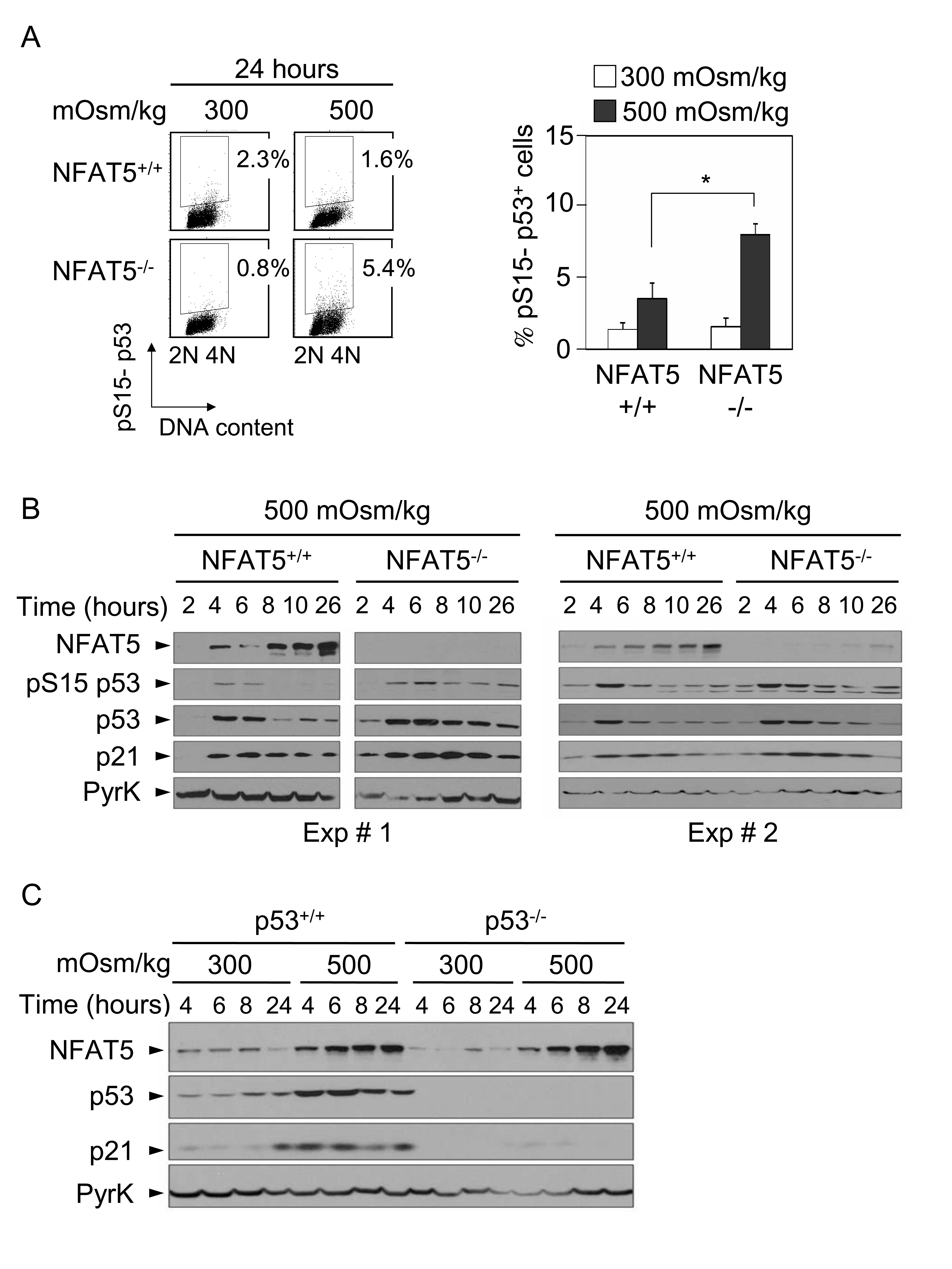

Supplement: Figure S4 — Induction of p53 and p21 in NFAT5−/− T cells in response to hypertonicity. A) Phospho-p53 (Ser15) was detected by intracellular staining in NFAT5+/+ and NFAT5−/− T cells cultured in isotonic or hypertonic medium during 24 hours. Results correspond to cells gated as alive. Dot plots show one representative experiment. Bars on the right represent the mean±SEM of four independent experiments (* = p<0.05). B) Time course of p53-Ser15 phosphorylation, accumulation of total p53 and p21 in NFAT5+/+ and NFAT5−/− T cells in response to hypertonicity were analyzed by Western blot. Pyruvate kinase (PyrK) is shown as protein loading control. C) Time course of NFAT5 and p21 induction in p53+/+ and p53−/− T cells in response to hypertonicity. The experiment shown is representative of three independently performed. (1.90 MB TIF) [file pone.0005245.s004.tif]

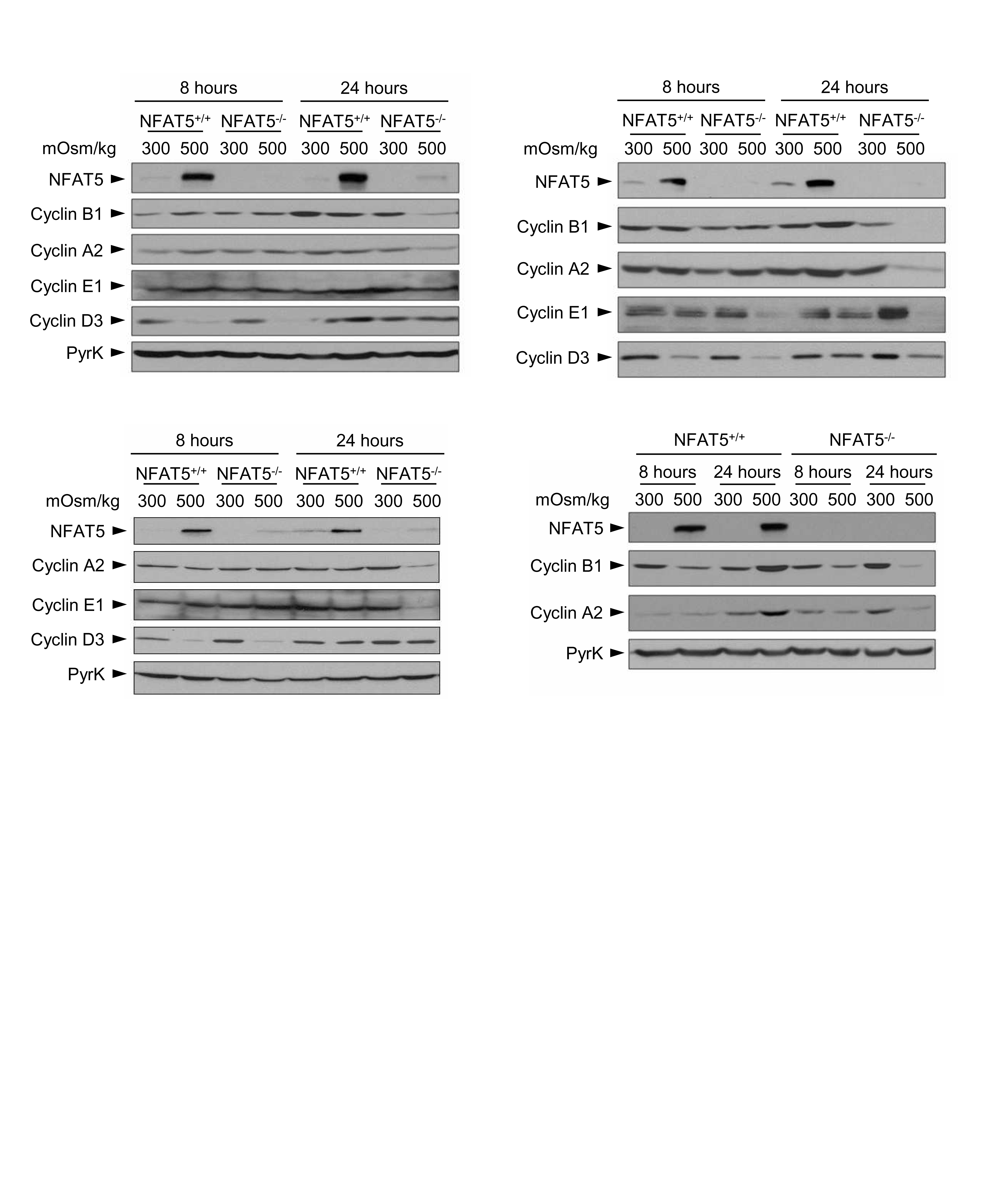

Supplement: Figure S5 — Expression of cyclins in proliferating NFAT5−/− T cells upon exposure to hypertonic conditions. Expression of cyclins D3, E1, A2 and B1 was analyzed by Western blot in lysates of proliferating NFAT5+/+ and NFAT5−/− T cells after 8 and 24 hours of hypertonicity treatment. Pyruvate kinase (PyrK) is shown as protein loading control. (1.81 MB TIF) [file pone.0005245.s005.tif]

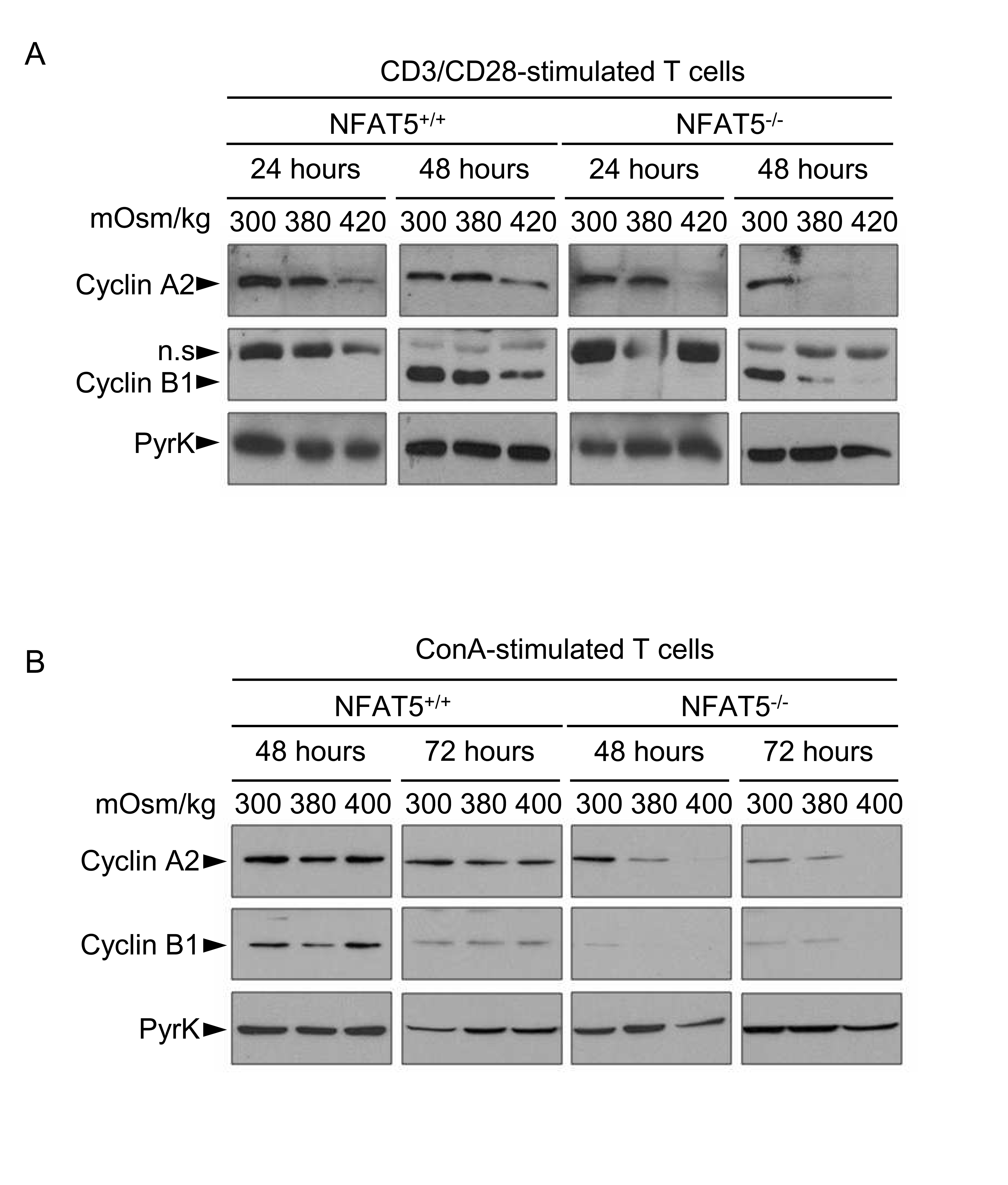

Supplement: Figure S6 — Effect of hypertonicity on cyclin induction by mitogens or T cell receptor activation in NFAT5−/− T cells. Expression of cyclins A2 and B1 was analyzed by Western blot in lysates of NFAT5+/+ and NFAT5−/− T lymphocytes induced to proliferate with (A) anti-CD3/CD28 antibodies plus IL-2 or (B) concanavalin A (ConA) plus IL-2 in isotonic or moderately hypertonic media. Pyruvate kinase (PyrK) was used as protein loading control. (2.20 MB TIF) [file pone.0005245.s006.tif]
